# DNA methylation of the *LIN28* pseudogene family

**DOI:** 10.1186/s12864-015-1487-3

**Published:** 2015-04-11

**Authors:** Aaron P Davis, Abby D Benninghoff, Aaron J Thomas, Benjamin R Sessions, Kenneth L White

**Affiliations:** Department of Animal, Dairy and Veterinary Sciences, Utah State University, 4815 Old Main Hill, Logan, UT 84322-4815 USA; School of Veterinary Medicine, Utah State University, Logan, UT USA; USTAR Applied Nutrition Research, Utah State University, Logan, UT USA

**Keywords:** DNA methylation, Pseudogene, *LIN28*

## Abstract

**Background:**

DNA methylation directs the epigenetic silencing of selected regions of DNA, including the regulation of pseudogenes, and is widespread throughout the genome. Pseudogenes are decayed copies of duplicated genes that have spread throughout the genome by transposition. Pseudogenes are transcriptionally silenced by DNA methylation, but little is known about how pseudogenes are targeted for methylation or how methylation levels are maintained in different tissues.

**Results:**

We employed bisulfite next generation sequencing to examine the methylation status of the *LIN28* gene and four processed pseudogenes derived from *LIN28*. The objective was to determine whether *LIN28* pseudogenes maintain the same pattern of methylation as the parental gene or acquire a methylation pattern independent of the gene of origin. In this study, we determined that the methylation status of *LIN28* pseudogenes does not resemble the pattern evident for the *LIN28* gene, but rather these pseudogenes appear to acquire methylation patterns independent of the parental gene. Furthermore, we observed that methylation levels of the examined pseudogenes correlate to the location of insertion within the genome. *LIN28* pseudogenes inserted into gene bodies were highly methylated in all tissues examined. In contrast, pseudogenes inserted into genomic regions that are not proximal to genes were differentially methylated in various tissue types.

**Conclusions:**

Our analysis suggests that *Lin28* pseudogenes do not aquire patterns of tissue-specific methylation as for the parental gene, but rather are methylated in patterns specific to the local genomic environment into which they were inserted.

**Electronic supplementary material:**

The online version of this article (doi:10.1186/s12864-015-1487-3) contains supplementary material, which is available to authorized users.

## Background

DNA methylation controls diverse aspects of genome regulation and transcriptional activity. Methylation of mammalian DNA involves the addition of a methyl group to the 5’-carbon of the cytosine in a cytosine-guanine (CpG) dinucleotide. This system of methylation likely evolved from a genomic defense system responsible for preventing the spread of parasitic genetic elements. DNA methylation has since evolved to play an active role in maintaining genetic structure and genome regulation [1]. Methylation is involved in X-chromosome inactivation [2,3], silencing of transposable elements [4-7], tissue-specific gene expression [8-11], and gene imprinting [12-15]. DNA methylation is widespread throughout the genome, and the maintenance of methylation patterns is highly regulated and tissue-specific [16,17]. DNA methyltransferases (DNMTs) are responsible for *de novo* and maintenance methylation of the genome [18-20]. The proper establishment and maintenance of methylation patterns is critical for early development and the absence of DNA methylation results in embryonic lethality [21-23].

DNA methylation also regulates pseudogenes within the genome [16,24]. Pseudogenes are decayed copies of active genes that have arisen from either a duplication event, in which the entire gene or portion of a gene is duplicated (non-processed pseudogenes), or from the retrotransposition of an RNA transcript into the genome (processed pseudogenes). An analysis of the human genome estimates that as many as 19,000 pseudogenes are evenly distributed throughout the genome, and approximately 70% of these are processed pseudogenes [20,25]. Ten percent of genes within the human genome have at least one corresponding pseudogene [20,26], and pseudogenes primarily arise from parental genes that are transcriptionally active within the germ line.

The same regulatory network that inhibits transposable element movement likely induces DNA methylation on pseudogenes. Methylation of pseudogenes is elevated in embryos, likely as a mechanism for preventing the spread of transposable elements during embryogenesis [16,27]. In plants, the inactivation of methyltransferases resulted in the widespread activation of transposable elements and pseudogenes [27], demonstrating that DNA methylation is sufficient to prevent the activation of pseudogenes. In humans, pseudogenes are highly methylated, presumably to prevent transcription and further transposition [16].

Characterizing methylation patterns of pseudogenes is critical, as pseudogenes with high sequence identity to parental genes can lead to misinterpretation of results in methylation studies [28,29]. The characterization of pseudogene methylation signatures also reveals how DNA segments are regulated by methylation networks once inserted into the genome. In order to better understand how methylation patterns are established and maintained on pseudogenes, we examined the methylation status of four pseudogenes derived from the translational enhancer *LIN28*. This gene is important in early embryo development and can also act as a reprogramming factor in the production of induced pluripotent stem cells [30,31].

*LIN28* has given rise to at least ten processed pseudogenes within the bovine genome that vary in length between 100 to 4000 bp (Btau_4.6.1, released Nov 2, 2011). The protein-coding region of *LIN28* contains a high concentration of CpG sites, making the gene a potential target for DNA methylation once inserted elsewhere in the genome as a pseudogene. By measuring the methylation levels of selected pseudogenes and the *LIN28* gene, we sought to determine whether the same regulatory mechanism that directs and maintains methylation of the *LIN28* gene also controls the methylation status of *LIN28* pseudogenes. Additionally we examined the expression of genes near the insertion site to determine whether pseudogene methylation is involved in transcriptional control of adjacent genes. This study is the first to characterize the methylation status of *LIN28* and its associated pseudogenes, and is the first such research to characterize the methylation status of a pseudogene family within the bovine genome.

## Results

**Figure 1:**
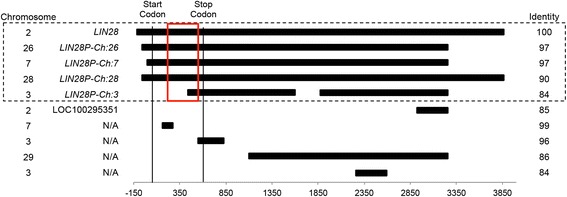
The nine processed pseudogenes of *LIN28* aligned to the *LIN28* processed transcript. The horizontal filled in lines represent each pseudogene with the location relative to the start of the *LIN28* protein-coding region indicated at the bottom and measured in base pairs. Vertical lines represents start and stop codons. Sequence identity of each pseudogene to the *LIN28* parental transcript is indicated on the right column and the chromosome of insertion is indicated on the left. The dashed box shows the pseudogenes examined for methylation analysis, and the red box indicates the location of CpG sites measured for this study.

**Figure 2:**
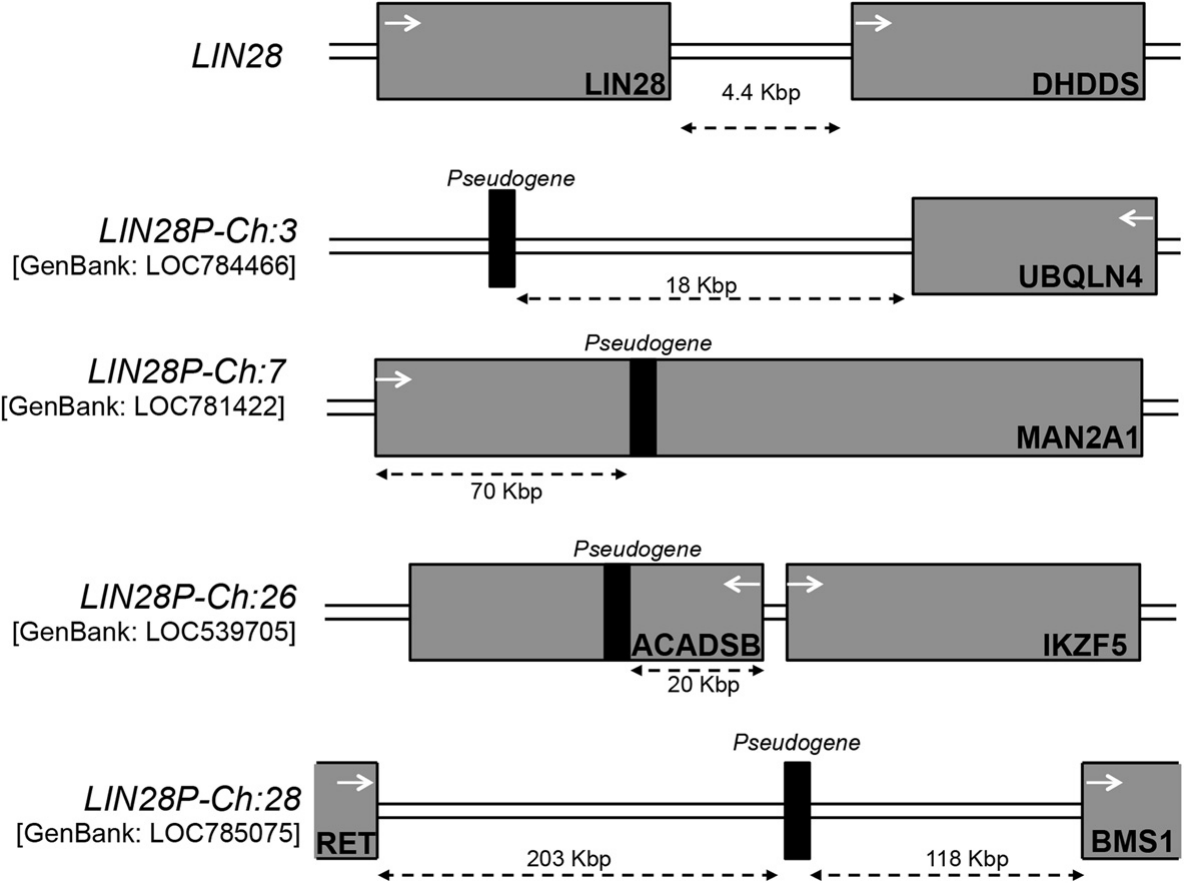
Location of *LIN28* pseudogenes on each chromosome. Genes are represented by gray boxes, while the locations of pseudogene insertion on the chromosome are indicated by the black boxes. White arrows indicate direction of gene transcription, and dashed arrows indicate the distance of each pseudogene relative to local genes. See Additional file 1: Figures S1-S4 for graphical representation of the sequence alignments of each pseudogene to *LIN28* and the location of all CpG sites examined.

**Figure 3:**
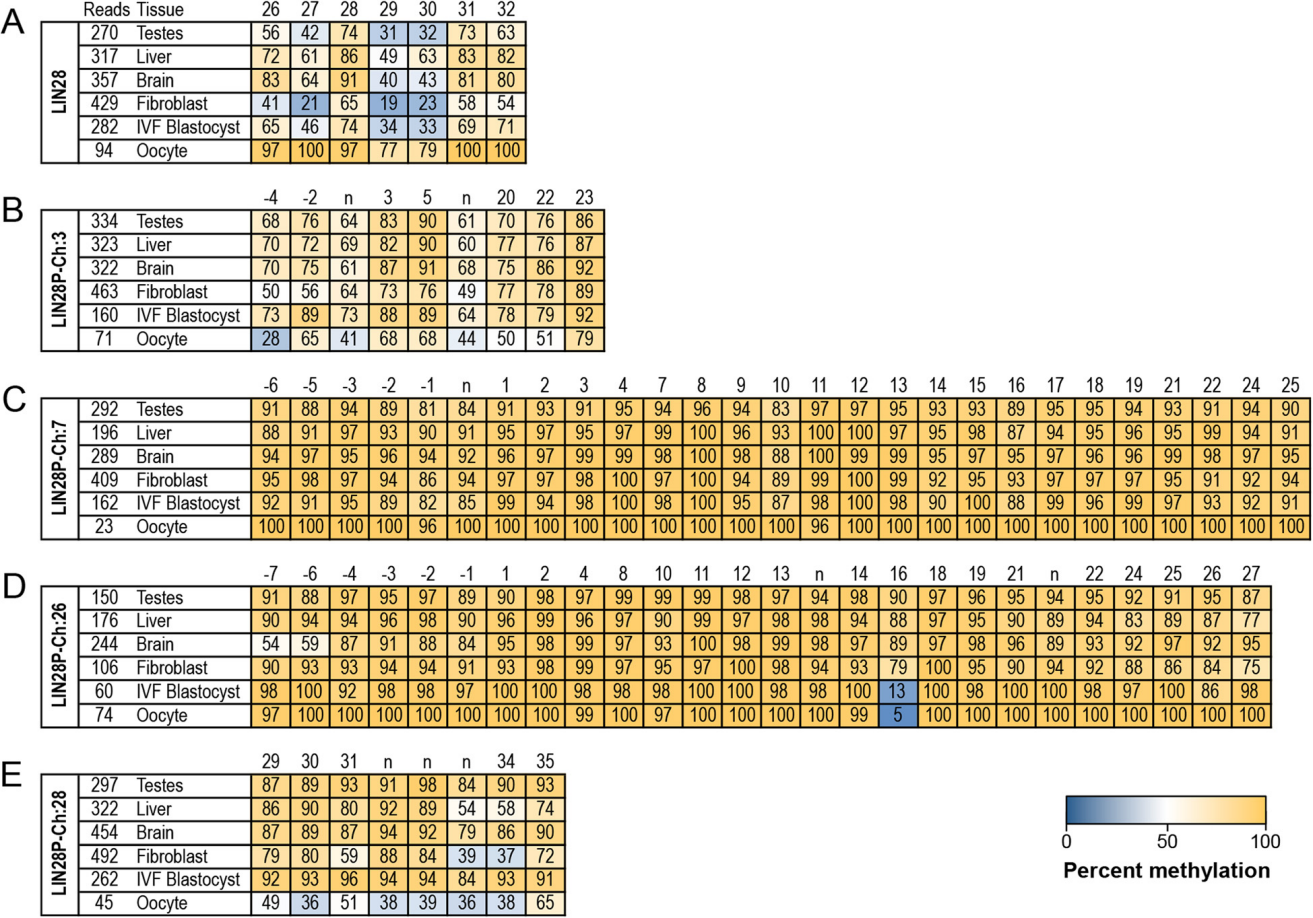
Heat map of *LIN28* and *LIN28* pseudogene methylation profile. Methylation profiles for *LIN28* (**A**) and pseudogenes, *LIN28P-CH:3* (**B**), *LIN28P-Ch:7* (**C**), *LIN28P:26* (**D**), and *LIN28P-CH:28* (**E**), are shown for the examined tissues: testes, liver, brain, fibroblast, IVF blastocyst embryos, and oocytes. The total number of sequencing reads is indicated next to each sample. The average percentage methylation of CpG sites within the entire amplicon is shown for each pseudogene. Each heat map cell indicates the percentage of CpG sites positive for methylation. Yellow indicates high methylation and blue low methylation. Each CpG site corresponds to a number indicated above each sample that indicates the placement of each CpG site in relation to the protein-coding region. CpG sites of pseudogenes that do not correspond to a CpG location within the *LIN28* parental gene are represented by ‘n’. See Additional file 1: Figures S1-S4 for placing these CpG sites in sequence context for each of the pseudogenes. See Additional file 1: Figures S5-S9 for heat map representations of methylation for all sequence reads across samples for each pseudogene.

We assessed the methylation status of four *LIN28* processed pseudogenes in six bovine tissue samples, including brain, liver, testes, fibroblast cells, IVF blastocyst stage embryo, and oocyte. Three of the *LIN28* pseudogenes contain the entire protein-coding sequence as well as a long region downstream from the stop codon. A fourth pseudogene contains only the terminal portion of the protein-coding region and downstream transcript (Figure 1; Additional file 1: Figures S1-S4). These pseudogenes are likely the products of retrotransposition of the *LIN28* transcript following RNA splicing. These four pseudogenes were selected for examination based on their retention of the protein-coding region, as well as insertion location and number of CpG sites. The pseudogenes examined were as follows: *LIN28P-Ch:3* [GenBank:LOC784466] inserted into chromosome 3 approximately 20Kbp upstream of the UBQLN4 gene (84% identity with *LIN28*), *LIN28P-Ch:7* [GenBank:LOC781442] on chromosome 7 inserted into the fourth intron of the *MAN2A1* gene (97% identity with *LIN28*), *LIN28P-Ch:26* [GenBank:LOC539705] on chromosome 26 inserted within the first intron of *ACADSB* (97% identity with *LIN28*), and *LIN28*P-*Ch28* [GenBank:LOC785075] on chromosome 28 has no proximity to any gene (90% identity with the *LIN28* gene) (Figure 2). Primer sets were designed to profile each individual pseudogene for a sequence within the protein-coding region. We selected a region that offered the greatest degree of overlap between the *LIN28* gene and associated pseudogenes. However, due to the high sequence identity between the pseudogenes and the parental gene, the overlap of examined CpG sites was limited by our ability to specifically amplify each individual pseudogene. The available sites in the LIN28 gene were restricted to just seven sites that were proximal to the overlapped regions within the pseudogenes. Pseudogenes *LIN28P-Ch:3*, *LIN28P-Ch:7* and *LIN28P-Ch:26* all contain considerable overlap between identical CpG sites. *LIN28P-Ch:26* contains overlap of only two CpG sites with the *LIN28* gene. The *LIN28* gene also shares only three overlapping CpG sites with *LIN28P-Ch:28*. Because of the restricted overlap of CpG specific sites and limited number of *LIN28* CpG sites, our assessment regarding differences between individual CpG dinucleotides among the four pseudogenes was limited. Rather we focused our analysis to consider patterns of methylation among the amplified regions from each pseudogene as a whole (Figure 3; Additional file 1: Figures S5-S9).

### Methylation status of the LIN28 family

Our results indicate that *LIN28* and all examined pseudogenes were methylated in all bovine cell and tissue types examined. Due to the sequence similarity between *LIN28* and its derived pseudogenes we were only able to obtain coverage of seven CpG dinucleotides within the *LIN28* gene. This region was selected because of primer-specific amplification adjacent to the region examined in the pseudogenes. These dinucleotides are located in the third exon of *LIN28*. All of the CpG sites were methylated in each tissue to varying degrees (Figure 3; Additional file 1: Figures S5-S9). In addition, all pseudogenes examined were methylated in all tissue samples. However, each pseudogene demonstrated a distinct methylation pattern that differed sharply from the *LIN28* gene, and that correlates with the pseudogene insertion location.

The pseudogenes *LIN28P-Ch:3* and *LIN28P-Ch:28* were inserted into a location isolated from any gene, and both of these pseudogenes share a similar overall pattern of methylation (Figure 3B, E; Additional file 1: Figures S6, S9). We analyzed methylation at nine CpG dinucleotides within *LIN28P-Ch:3* and eight CpG dinucleotides in *LIN28P-Ch:28*. In both pseudogenes, oocytes had the lowest levels of overall methylation and blastocysts had the highest level of methylation. The methylation patterns of both pseudogenes apparently deviated from patterns observed in the *LIN28* gene. Within the *LIN28* gene, oocytes had the highest frequency of methylation. Alternatively, the methylation of the parental gene was lowest in blastocyst embryos. In *LIN28P-Ch:28* we observed three overlapping CpG dinucleotides with the *LIN28* gene. Although limited, within these three overlapping sites, the inverse pattern of methylation between the *LIN28* gene and pseudogenes *LIN28P-Ch:3* and *LIN28P-Ch:28* is maintained.

In contrast to the tissue-specific methylation patterns, pseudogenes *LIN28P-Ch:7* and *LIN28P-Ch:26* both are highly methylated (Figure 3C-D; Additional file 1: Figures S7-S8). Both *LIN28P-Ch:7* and *LIN28P-Ch:26* were inserted into gene introns. *LIN28P-Ch:7* is inserted into the fourth intron of the gene *MAN2A1*. A majority of the 27 CpG sites inspected within this pseudogene were methylated in all tissue samples (Figure 3C; Additional file 1: Figure S7). The *LIN28P-Ch:26* pseudogene lies within the first intron of the gene *ACADSB* and is 20 kbp from the gene *IKZF5*. Similar to *LIN28P-Ch:7,* all 26 CpG sites examined for the *LIN28P-Ch:26* pseudogene were highly methylated at a high frequency (Figure 3D; Additional file 1: Figure S8). The high levels of methylation observed in both of these pseudogenes is in sharp contrast to the moderate levels of methylation of the *LIN28* gene and pseudogenes not inserted into gene bodies, none of which are as highly methylated for all tissue samples.

These apparent differences in methylation correlate to the location of insertion of the pseudogene. *LIN28* pseudogenes inserted into gene bodies were highly methylated. Alternatively, *LIN28* pseudogenes with an insertion location distant from a gene varied in the pattern of methylation in a tissue-specific manner, although these tissue-specific patterns were inverse with respect to the *LIN28* gene (Figure 3; Additional file 1: Figures S5-S9).

### Non-CpG methylation

We observed two cytosine nucleotides that were not contained within CpG dinucleotides that were methylated to some degree in all tissues examined. Both cytosine nucleotides were located within *LIN28P-Ch:7.* Both non-CpG methylation sites were located within close proximity to CpG sites. One non-CpG methylated cytosine was located between CpG sites three and four (CG**C**CG) on *LIN28P-Ch:7*. The percentage of sequences that were methylated at this location in the tissues examined were: 9% in testes, 13% in liver, 20% in brain, 46% in fibroblast, 46% in IVF blastocyst, and 4% in oocyte. Both CpG sites flanking the specified cytosine are highly methylated in all samples (Figure 3C; Additional file 1: Figure S7). The second non-CpG methylated cytosine observed is upstream from CpG site 13 in *LIN28P-Ch:7* (**C**CG). The percentage of methylated sequences at this cytosine were observed to be: 8% in testes, 10% in liver, 16% in brain, 38% in fibroblast, 40% in IVF blastocyst, and 100% in oocyte. Methylation of CpG site 13 on *LIN28P-Ch:7* is high in all tissue samples (Figure 3C; Additional file 1: Figure S7). Additionally, the methylation status of either non-CpG cytosine nucleotides is independent of the other.

### Single CpG demethylation

*LIN28P-Ch:26* contains a single CpG dinucleotide that is characterized by a low frequency of methylation in a tissue-specific manner. This CpG dinucleotide is under-methylated in a tissue-specific manner at CpG site 16 in IVF blastocyst and oocyte samples (Figure 3D; Additional file 1: Figure S8). Both samples are also under-methylated relative to adjacent CpG sites with a 13% methylation frequency in IVF blastocyst and 5% frequency in oocytes, yet highly methylated for other tissue types.

### Expression of genes adjacent to LIN28 pseudogenes

**Figure 4:**
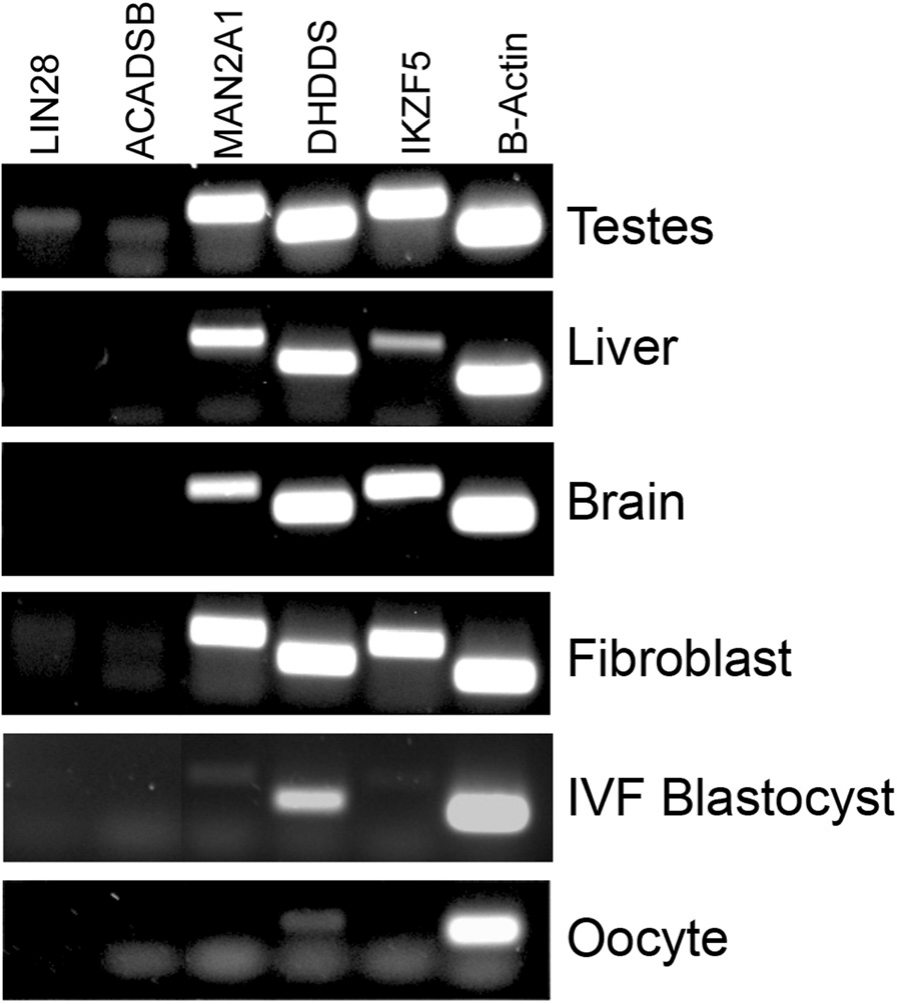
End-point RT-PCR. Expression of genes *LIN28*, *ACADSB*, *MAN2A1*, *DHDDS*, *IKZF5* and *ACTB* in the testes, liver, brain, fibroblast, IVF blastocyst embryos and oocytes.

End-point RT-PCR was performed to determine gene expression of *LIN28* as well as genes closely adjacent to the four *LIN28* pseudogenes in order to establish whether methylation of pseudogenes correlated with differences in gene expression. Expression of the genes *DHDDS*, *MAN2A1*, *ACADSB,* and *IKZF5* were measured in testes, liver, brain, fibroblast, IVF blastocyst, and oocyte samples (Figure 4). Gene expression of all genes examined did not correspond to methylation patterns of adjacent pseudogenes (Figures 3 and 4; Additional file 1: Figures S5-S9). *IKZF5* is proximal to *LIN28P-Ch:26,* which does contain a tissue-specific methylation pattern of a single CpG site. Interestingly, the expression pattern for *IKZF5* mirrors the pattern of methylation for *LIN28P-Ch:26* in that this gene is not expressed in oocytes or blastocyst embryos (corresponding to samples with low methylation at site 16), but is highly expressed in other tissue types (corresponding to samples with high methylation at site 16) (Figures 3 and 4; Additional file 1: Figures S5-S9).

## Discussion

The objective of our study was to determine whether the methylation of pseudogenes followed the same patterns as the functional parental gene by examining *LIN28* as a case study. An observation that the *LIN28* pseudogenes maintained an identical or highly similar methylation pattern as the *LIN28* gene would indicate that regulation of *LIN28* methylation is intrinsic to the gene sequence and that methylation of pseudogenes with high sequence identity is likely controlled by the same mechanism(s) that maintain methylation of the *LIN28* gene. Alternatively, an observation that pseudogene methylation patterns deviated from the parental gene would indicate that pseudogenes are subject to local regulation of methylation patterns. In this study, we observed that *LIN28* pseudogenes do not recapitulate the same methylation status as *LIN28*, but rather appear to acquire methylation patterns independent of the parental gene. Furthermore, we observed that methylation levels of the examined pseudogenes correlate to the location of insertion. To our knowledge, this study is the first to characterize the methylation signatures of the *LIN28* pseudogene family and to identify an effect of genome location on pseudogene methylation. Previous studies have shown that methylation patterns of pseudogenes deviate from those of the parental gene, and our findings are consistent with these observations [16,27,29,32].

Our examination of an entire pseudogene family highlights the diversity of methylation patterns apparent for highly similar sequences. *LIN28* pseudogenes inserted into gene bodies were highly methylated in all tissues examined. In contrast, pseudogenes inserted into genomic regions that are not proximal to genes had reduced overall methylation and were differentially methylated in unique tissue types. The measurement of methylation patterns in six distinct tissue types showed that methylation of pseudogenes can be highly variable in different tissues (Figure 3; Additional file 1: Figures S5-S9) and suggested that methylation of CpG sites may be differentially regulated in these tissues. Pseudogenes not associated with genes had less methylation in tissue samples that were highly methylated in the parental gene.

We measured methylation of seven CpG dinucleotides located within the third exon of the *LIN28* gene. Methylation of CpG dinucleotides within the gene body is generally associated with transcriptionally active genes [25,26]. This pattern is in contrast to methylation of the 5’ upstream and promoter regions of genes, which are typically associated with transcriptional silencing. Methylation of these seven CpG dinucleotides was dependent upon tissue type. Notably, oocytes had the highest levels of methylation within the *LIN28* gene. Oocytes generally maintain low levels of global methylation relative to somatic cells [33,34]. Following fertilization, global methylation levels decline further and are then reestablished during embryonic and somatic cell development [35-38]. The methylation level observed in the *LIN28* gene is counter to this pattern. However, methylation levels measured in the *LIN28P-Ch:3* and *LIN28P-Ch:28* pseudogenes are consistent with changes in methylation levels on a global level. Within both these pseudogenes, oocytes maintained low levels of methylation, whereas somatic cells maintained high levels of methylation. It is possible that the methylation patterns observed for *LIN28P-Ch:3* and *LIN28P-Ch:28* are maintained by the same mechanism that maintains global levels of methylation, while methylation of the *LIN28* gene is maintained by a separate mechanism.

We also observed that methylation of pseudogenes depends on the genomic context into which the pseudogene was inserted. Both pseudogenes inserted into the intron of a gene, *LIN28P-Ch:7* and *LIN28P-Ch:26*, were highly methylated in all tissues. The absence of variability among tissues is revealing, as insertion of a pseudogene into a gene body appears to induce high levels of methylation. On the other hand, both pseudogenes not associated with a gene, *LIN28P-Ch:3* and *LIN28P-Ch:28*, vary in the pattern of methylation within different tissue samples, but tissue methylation levels were similar between the two pseudogenes. The similarity of methylation levels among all tissue types between similar pseudogenes indicates that pseudogenes share a common regulatory mechanism that establishes and maintains the methylation signature. When considering the location of the *LIN28* pseudogenes in context of chromosomal structure, we originally postulated that *LIN28P-Ch:7* and *LIN28P-Ch:26* exist in a region that is likely euchromatin, as both pseudogenes were inserted into gene bodies (Figure 2). Furthermore, expression data obtained for *MAN2A1* and *IKZF5* (Figure 4) suggest that these regions are transcriptionally active, providing evidence that the region maintains euchromatin structure in the tissues examined. Because *LIN28P-Ch:3* and *LIN28P-Ch:28* are isolated from any local gene, it is reasonable to expect that the local sequence environment for these genes is heterochromatin in structure. However, our expression analysis did not directly test this hypothesis, since these two pseudogenes do not lie within a gene body.

Other researchers have shown that a limited number of pseudogenes have evolved to play a regulatory function within the genome [39-44]. It is possible that the introduction of a pseudogene within a the promoter of a gene may provide a CpG-rich sequence that can be utilized as a site for gene silencing via DNA methylation, although this observation was not evident in our study. As indicated by the results of our analysis of *LIN28*, these pseudogenes may undergo tissue-specific methylation unique to the region of pseudogene insertion, which lead us to investigate the relationship between methylated pseudogenes and expression of adjacent genes. However, evidence from PCR analysis of genes associated with the four pseudogenes, including *DHDDS*, *MAN2A1*, *ACADSB*, and *IKZF5*, suggested that *LIN28* pseudogene methylation status was not correlated with decreased gene expression. This observation suggests that in the case of the *LIN28* pseudogene family, methylation status of the pseudogene does not play a major role in regulation of expression of the local genes. However, methylation of a single CpG dinucleotide in the *LIN28* pseudogene was correlated with gene expression changes for the gene *IKZF5*. The *LIN28P-Ch:26* pseudogene is located within the first intron of *ACADSB* and 20 Kbp upstream from *IKZF5*. Within the *LIN28P-Ch:26* pseudogene, a single CpG dinucleotide at site 16 is hypomethylated in the oocyte and blastocyst samples, a sharp contrast to the hypermethylated status of all surrounding CpG dinucleotides as well as the same CpG site in the four remaining tissues. Interestingly, *IKZF5* was not expressed in either oocytes or blastocyst embryos. Although the expression pattern of *IKZF5* was correlated with the tissue-specific methylation patterns observed for the pseudogene, it is doubtful that this single CpG site is involved in the transcriptional regulation of *IKZF5*.

Within *LIN28P-Ch:7*, we identified two cytosine nucleotides that are methylated outside of the CpG dinucleotide context. In both cases, the methylation has occurred on a cytosine immediately upstream from a CpG dinucleotide. Non-CpG methylation has been observed to occur with higher frequency in non-dividing cells and gametes, although its function remains unknown [45]. Interestingly, the same cytosine nucleotides are observed within the *LIN28P-Ch:26* and are unmethylated in all samples. This observation further suggests that upon integration into the genome, methylation of the *LIN28* pseudogene family is location specific and the methylation occurs independent of their sequence.

## Conclusions

In conclusion, the four *LIN28* pseudogenes examined in this study maintained methylation patterns that deviate from those found in the parental gene as well as from one another according to the genomic location into which the pseudogene was inserted. To our knowledge, this is a unique phenomena identified in our study, and examination of more pseudogene families will be required to determine whether the same observation is consistent for other integrated pseudogenes. Pseudogenes derived from *LIN28* have undergone mutations and no longer maintain an exact sequence identity to the *LIN28* gene. Sequence identity ranges from 84% to 97% (Figure 1), and it is possible that the differences in methylation pattern are a result of changes in the pseudogene sequence. Additionally, our conclusions are based on methylation of CpG sites within the pseudogenes only, as well as a limited number of CpG sites within the *LIN28* gene. Analysis of CpG dinucleotides throughout the *LIN28* parent gene as well as CpG dinucleotides flanking the pseudogene point on insertion would further add to our findings. Future work should focus on these CpG sites and would further help determine how DNA methylation is targeted to specific genomic regions. Although methylation status of the *LIN28* pseudogenes was not associated with changes gene expression of proximal genes, this observation does not rule out the possibility that CpG-rich pseudogenes could serve as sites for regulation of gene expression by methylation, a hypothesis that may also be addressed by survey of other pseudogene families. New knowledge on the regulation of pseudogenes via DNA methylation could contribute to greater understanding of the maintenance of global and/or regional patterns of methylation. Future work on this topic should focus on characterizing methylation patterns for other pseudogene families to determine whether all pseudogenes are maintained in a similar manner or whether sequence specific patterns can be identified through analysis of pseudogenes.

## Methods

### Fibroblast cell culture

Bovine fibroblasts isolated from skin were cultured in DMEM F12 (Thermo Scientific HyClone Laboratories, Logan, UT) supplemented with 15% fetal bovine serum (FBS) (Thermo Scientific HyClone Laboratories), 100 U/ml penicillin, and 100 mg/ml streptomycin. Cells were cultured at 37°C with 5% CO_2_. For cell collection fibroblasts were treated with 0.25% trypsin prior to collection for RNA and DNA isolation.

### Oocyte maturation

The Utah State University Institutional Animal Care and Use Committee approved all procedures for the use of animals in this study (protocol #1506). Bovine ovaries were collected at a local abattoir (E.A. Miller, Hyrum, UT) and used for collecting oocytes. Oocytes with 3 to 8 mm follicles were aspirated along with cumulus complexes. Following aspiration, cumulus oocyte complexes were cultured at 37°C with 5% CO_2_ for 18 to 22 hr in TCM 199 maturation medium containing 10% FBS (Thermo Scientific HyClone Laboratories), 0.05 mg/ml follicle stimulating hormone, 5 mg/ml luteinizing hormone, 100 U/ml penicillin, and 100 mg/ml streptomycin.

### In vitro fertilization

Following 18 to 22 hr of oocyte maturation, cryopreserved bovine semen (Hoffman AI, Logan, UT) was thawed in a 37°C water bath. Live sperm were isolated by centrifugation through a 45%/90% Percoll® gradient. Sperm were suspended in Tyrode’s albumin lactate pyruvate (TALP) and used for oocyte fertilization. Twenty-four hr post fertilization, cumulus cells were removed by vortexing the cumulus oocyte complex in phosphate buffered saline (PBS) containing 0.32 mM sodium pyruvate, 5.55 mM glucose, 3 mg/ml BSA, and 10 mg/ml hyaluronidase. Oocytes were washed through six drops of PBS and placed in co-culture dishes plated with cultured cumulus cells and cultured in CR2 medium.

### Tissue collection and RNA isolation

Twenty-five pooled blastocyst embryos were collected after 8 days of culture in CR2 medium, snap frozen with liquid nitrogen, and stored at -80°C. Twenty-five pooled oocytes were collected after 22 hr of maturation, vortexed for 5 min in PBS to remove cumulus cells, snap frozen with liquid nitrogen, and stored at -80°C. RNA was isolated from oocyte and blastocyst embryo samples using the RNeasy Mini Kit (Qiagen, Germantown, MD) following manufacturer’s instructions. Bovine brain, liver, testes tissue samples were collected immediately after slaughter and suspended in RNALater (Ambion, Austin, TX). Samples were stored overnight at 4°C. Fibroblasts were collected as described above. RNA was isolated from brain, liver, testes, and fibroblasts using TRIzol reagent (Life Technologies, Carlsbad, CA). Tissue samples were homogenized in 3 ml TRIzol with a tissue homogenizer. Cells were incubated for 5 min at room temperature, combined with 0.6 ml chloroform, and mixed by inversion. Samples were centrifuged at 12,000 × g for 15 min at 4°C, the upper aqueous phase was removed and combined with 1.5 ml isopropyl alcohol, then centrifuged at 12,000 × g for 10 min at 4°C. Supernatant was removed and the RNA was washed with 75% ethanol and centrifuged again, then dried and resuspended in H_2_O. Isolated RNA was immediately converted to cDNA using the Superscript III Reverse Transcriptase Kit (Life Technologies) following manufacturer’s protocol. Samples were stored at -20°C until use.

### DNA isolation and bisulfite conversion

Twenty-five pooled oocytes and blastocyst embryos were snap frozen with liquid nitrogen and stored at -80°C until direct bisulfite conversion. DNA from brain, liver, testes, and fibroblast samples were collected from the interphase of the TRIzol treatment used for RNA collection. The collected interphase was combined with 0.9 ml ethanol, mixed by inversion, and centrifuged at 2000 × g for 5 min at 4°C. The pellet was suspended with 75% ethanol and washed by centrifugation three times. The DNA pellet was resuspended in 0.1 μM sodium citrate. The isolated DNA underwent bisulfite conversion using the EZ DNA Methylation Kit (Zymo Research, Irvine, CA) according to manufacturer’s recommendation. DNA was stored at -20°C until use. Bisulfite PCR and 454 sequencing

Primers for bisulfite-converted DNA were designed for each pseudogene and the *LIN28* gene (Table 1). Primers covered CpG sites within and immediately surrounding the protein-coding sequence of *LIN28*. As the four examined pseudogenes maintain high sequence identity to the *LIN28* gene and one another, primers were designed specific to each pseudogene to ensure only amplification of the target pseudogene. Bisulfite-converted DNA was used as a template for 25 μl PCR reactions using 1 to 3 μl DNA. Reactions were carried out using the Mastercycler thermal cycler (Eppendorf, New York, NY). Primer concentrations were 0.6 μM for all reactions. All reactions were carried out using the following cycling parameters: 30 cycles of 94°C for 1 min, annealing temperature ranging from 54 to 59°C for 1 min, and 72°C for 1 min for 30 cycles. A second PCR was performed using primers including the original primer sequence with the addition of the 454 adapter sequence, key sequence, and molecular identification tags (designated as N) to differentiate individual tissue samples (adapter A CGTATCGCCTCCCTCGCGCCATCAGNNNNNNNNNN attached to the forward primer and adapter B CTATGCGCCTTGCCAGCCCGCTCAGNNNNNNNNNN attached to the reverse primer). The following cycling parameters were used: 94°C for 30 sec, annealing temperature ranging from 55 to 60°C for 30 sec, and 72°C for 30 sec for 15 cycles. The second reaction used 1 μl of PCR product from the first PCR and 0.3 μM primer. All PCR samples used GoTaq Green Master Mix (Promega, Madison, WI). All PCR reactions were carried out on a Mastercycler thermal cycler (Eppendorf). PCR reactions were purified with AMPure beads (Beckman Coulture, Brea, CA) and quantified using PicoGreen (Life Technologies). PCR product was sequenced using the 454 GD FLX Titanium DNA sequencer (Roche, Indianapolis, IN). Amplicon libraries were subjected to emulsion PCR to generate DNA-coated beads, loaded onto a PicoTiterPlate, and sequenced with a FLX Titanium DNA sequencing Kit according to the manufacturer’s protocol.

**Table 1:** **Primers for bisulfite sequencing**

*LIN28P-Ch:7*	Forward	GAAAAGTTTATGAAAGAGGTTAGGGTT
	Reverse	AAAACCCTCCATATACAACTTACTC
*LIN28P-Ch:28*	Forward	TTTTAAGAAGTTTATTAAGGGTTTGGAA
	Reverse	TAACCCCCACCCACTATAACTTAAT
*LIN28P-Ch:3*	Forward	GTATATAATGGGGAGTAGGGGTT
	Reverse	CAAAAACCCTCCAAATACAACTTAC
*LIN28*	Forward	TTTTTTTAGAGTAAGTTGTATATGGAGGG
	Reverse	TTACCAAAAACCACAAACTTCACTT

### End-point RT-PCR

End-point RT-PCR reactions targeting bovine cDNA were carried out using the Mastercycler thermal cycler (Eppendorf). GoTaq Green Master Mix (Promega) was used on all reactions with primer concentrations of 0.6 μM using the following cycling parameters: 40 cycles of 94°C 15 sec, annealing temperature ranging from 58 to 60°C for 15 sec, and 72°C for 30 sec for 35 cycles. Following cycling, samples were electrophoresed on a 1% agarose gel and imaged. Primers for end-point RT-PCR are shown in Table 2.

**Table 2:** **Primers for end-point RT-PCR**

*MAN2A1*	Forward	GACCCATTTGGACATTCACC
	Reverse	TTTAGGATCAGGCCCACAAG
*ACADSB*	Forward	TGGAAAACTCCTCCTCATGC
	Reverse	CAACTTGTTCCTGGGCAAAT
*IKZF5*	Forward	GAGAAGAAACCGGAGCCTTT
	Reverse	AGGTCCTTTCAAACCCGTCT
*DHDDS*	Forward	GTCATTTTGGGAGCGGTTCT
	Reverse	GCCAGTTTGTTGAAGCCTTG
*LIN28*	Forward	TGCAGAAACGCAGATCAAAG
	Reverse	TTCTTCCTCCTCCCGAAAGT
*ACTB*	Forward	ATGGGCCAGAAGGACTCGTA
	Reverse	CTTCTCCATGTCGTCCCAGT

### Sequence analysis

Sequencing data was analyzed to generate the frequency of methylation using BISMA analysis software [46]. Any sequence with a conversion rate less than 98% was excluded from sequence analysis. The average conversion rate of all sequences was 99.2%. Data are presented as the frequency of methylation on a per CpG site basis (number of transcripts with methylated CpG site ÷ total number of transcripts sequenced × 100). In order to account for the potential of a C-to-T transition causing misinterpretation of our results, known C/T SNPs were identified in the Single Nucleotide Polymorphism database (http://www.ncbi.nlm.nih.gov/snp). The database identified two CpG sites that exist as either a cytosine or thymine in normal populations. CpG site 23 (rs43296056) in *LIN28P-Ch:7* and CpG site 9 (rs208890804) in *LIN28P-Ch:26* (Additional file 1: Figures S2-S3). The identified sites were excluded from all methylation analysis.

## Additional file

Additional file 1:
**Supplemental figures, which include alignments for each of the pseudogenes with** ***LIN28*** **(Additional file 1: Figures S1-S4) and complete methylation data sets for all genes and tissues examined (Additional file 1: Figures S5-S9).**
