# Supplementary material for: DNA methylation of the LIN28 pseudogene family
Source: BMC Genomics. 2015 Apr 11;16(1):287. doi: 10.1186/s12864-015-1487-3 (PMC4404226; doi:10.1186/s12864-015-1487-3)
Supplement: Additional file 1: — Supplemental figures, which include alignments for each of the pseudogenes with LIN28 (Additional file 1: Figures S1-S4) and complete methylation data sets for all genes and tissues examined (Additional file 1: Figures S5-S9). [file 12864_2015_1487_MOESM1_ESM.pdf]

SUPPLEMENTAL FIGURES

```
                          -4   -3 -2        -1                            n
LIN28P-Ch3      AGCAGGGGCCCGGGGCTGCGGGCTCA-TAGAC---CATGAGCTCTGTGTCACACCACCGG
LIN28           AGCAGGGGCCCGGGGCCGCGGGCTCAGCCGACTACCATGGGCTCTGTGTCAAACCAGCAG
                **************** *********   ***   **** *********** **** * *

                  1           2       3   4       5   6     7 8   9 10 11 12
LIN28P-Ch3      TTTGCAGGTGGCTGCACCAAGGCGCCAGAGGAGGCGCCCAAGGA----------------
LIN28           TTCGCAGGTGGCTGCGCTAAGGCGCCGGAGGAGGCGCCGGAGGACGCGGCCCGCGCGGCG
                ** ************ * ******** ***********  ****

                       13           14   15                   16   17
LIN28P-Ch3      -----GCCACAGGTACTCCACAGTGCTGGCTTCTATGAATGCTTCAGTGTGTGCTTGGCA
LIN28           GAGGAGCCGCAGCTGCTGCACGGTGCGGGCATCTGTAAGTGGTTCAACGTGCGCATGGGG
                     *** *** * ** *** **** *** *** * * ** ****  *** ** ***

                  18 n                   20         2223  24      25
LIN28P-Ch3      TTTGGCGTCCTGTCCATGACCCACCCGGGTAGGGGTCGCGATCAGCCCTGCAGTGGATGT
LIN28           TTCGGCTTCCTGTCCATGACC-GCCCGCGCAGGGGTCGCGCTCGACCCCCCGGTGGATGT
                ** *** ************** **** * ********** **  ***  * ********

                                                     26
LIN28P-Ch3      CTCTGTGCACCAGAGCAAGCTGCACCTGGAGGGCTTCTG---------------------
LIN28           CTTTGTGCACCAGAGTAAGCTGCACATGGAGGGCTTCCGGAGCCTGAAGGAGGGGGAGGC
                ** ************ ********* *********** *

                27                      28                    29      30
LIN28P-Ch3      ------------------------------------------------------------
LIN28           CGTGGAGTTCACCTTTAAGAAGTCCGCCAAAGGCCTGGAATCTATCCGAGTCACCGGCCC

                                               31                         32
LIN28P-Ch3      ------------------------------------------------------------
LIN28           TGGGGGGGTGTTCTGTATTGGGAGTGAAAGGCGGCCCAAAGGGAAGAATATGCAGAAACG
```

Figure S1. Sequence alignment of *LIN28P-Ch:3* to the *LIN28* transcript. *LIN28P-Ch:3* sequenced region (top) is shown aligned to the matching region of the *LIN28* mRNA transcript (bottom). Matching nucleotides are indicated by a star below both nucleotides. CpG dinucleotides are highlighted in yellow on both sequences. Numbers above the pseudogene sequence indicates the placement of each CpG site in relation to the protein-coding region of the *LIN28* gene, with red numbers indicating the CpG sites analyzed in the pseudogene. CpG sites of pseudogenes that do not correspond to a CpG location within the *LIN28* parental gene are represented by 'n'. CpG dinucleotides highlighted in blue correspond to the CpG sites analyzed in the *LIN28* gene.

```
                          -6                     -5               -4    -3 -2
LIN28P-Ch7          GCCTCCGGACTTCTCTGGGGCCAGCAGCCGCCCAAGCAGGGGCCTGGGGCCGCGGGCTCA
LIN28               GCCTCCGGACTTCTCTGGGGCCAGCAGCCGCCCAAGCAGGGGCCCGGGGCCGCGGGCTCA
                    ******************************************** ***************

                      -1                     n         1           2      3  4
LIN28P-Ch7          GCCGACTACCATGGCTCTGTGTCCCACGAGCAGTTCGCAGGTGGCTGCGCTAAGGCGCC
LIN28               GCCGACTACCATGGCTCTGTGTCAAACCAGCAGTTCGCAGGTGGCTGCGCTAAGGCGCC
                    ***********************  ** ********************************

                    4       5  6    7 8    9 10 11 12      13           14
LIN28P-Ch7          GGAGGA------------CGCGGCCCGCGCGGCGGAGGAGCCGCAGATGCTGCACGGTGC
LIN28               GGAGGAGGCGCCGGAGGACGCGGCCCGCGCGGCGGAGGAGCCGCAGCTGCTGCACGGTGC
                    ******            **************************** *************

                    15                    16  17         18                19   20
LIN28P-Ch7          CGGCATCCCTAAGTGGTTCAACGTGCGCATGGGGTTCGGCTTCCTGTCCATGACCGCCAG
LIN28               GGGCATCTGTAAGTGGTTCAACGTGCGCATGGGGTTCGGCTTCCTGTCCATGACCGCCCG
                     ******  ************************************************* *

                    21      22 23 24       25
LIN28P-Ch7          CGCAGGGGTCGCGCTCGACCCCCCCGGTGGATGTCTTTGTGCAGCAGAGTAAGCTGCACA
LIN28               CGCAGGGGTCGCGCTCGACCCCCC-GGTGGATGTCTTTGTGCACCAGAGTAAGCTGCACA
                    ************************ ****************** ****************

                               26                     27                       28
LIN28P-Ch7          TGGAGGGCTTC-------------------------------------------------
LIN28               TGGAGGGCTTCCGGAGCCTGAAGGAGGGGGAGGCCGTGGAGTTCACCTTTAAGAAGTCCG
                    ***********

                                         29              30
LIN28P-Ch7          ------------------------------------------------------------
LIN28               CCAAAGGCCTGGAATCTATCCGAGTCACCGGCCCTGGGGGGGTGTTCTGTATTGGGAGTG

                         31                          32
LIN28P-Ch7          ------------------------------------------------------------
LIN28               AAAGGCGGCCCAAAGGGAAGAATATGCAGAAACGCAGATCAAAGGGAGACAGGTGCTACA
```

Figure S2. Sequence alignment of *LIN28P-Ch:7* to the *LIN28* transcript. *LIN28P-Ch:7* sequenced region (top) is shown aligned to the matching region of the *LIN28* mRNA transcript (bottom). Matching nucleotides are indicated by a star below both nucleotides. CpG dinucleotides are highlighted in yellow on both sequences. Numbers above the pseudogene sequence indicates the placement of each CpG site in relation to the protein-coding region of the *LIN28* gene, with red numbers indicating the CpG sites analyzed in the pseudogene. CpG sites of pseudogenes that do not correspond to a CpG location within the *LIN28* parental gene are represented by 'n'. CpG dinucleotides highlighted in blue correspond to the CpG sites analyzed in the *LIN28* gene. Site 23 was identified as a likely SNP (C>T conversion), and was thus excluded from further analysis.

```
                                                 -7                 -6
LIN28P-Ch26     ATTGTGCTGGGGAAGATGTAGCTGCCTGTTCCCCGAGCCACCCCTTTGCCTCCGGACTTC
LIN28           ATTGTGCTGGGGAAGATGTAGCTGCCTCTTCCCCGAGCCACCCCTTTGCCTCCGGACTTC
                *************************** ********************************

                                -5             -4   -3 -2        -1
LIN28P-Ch26     TCTGGGGCCAGCAGCCACCCAAGCAGGGGCCCGGGGCCGCGGACTCAGCCGACTACCATG
LIN28           TCTGGGGCCAGCAGCCGCCCAAGCAGGGGCCCGGGGCCGCGGGCTCAGCCGACTACCATG
                **************** ************************* *****************

                                       1           2        3 4        5  6
LIN28P-Ch26     GGCTCTGTGTCAAACCAGCAGTTCGCAGGTGGCTGCGCTAAGGTGCCGGAGGA-------
LIN28           GGCTCTGTGTCAAACCAGCAGTTCGCAGGTGGCTGCGCTAAGGCGCCGGAGGAGGCGCCG
                ******************************************* *********

                     7 8    9 10 11 12      13 n         14
LIN28P-Ch26     -----TGCGGCCCGCGCGGCGAAGGAGCCGCGGCTGCTGCACGGGGCAGGCATCTGTAAG
LIN28           GAGGACGCGGCCCGCGCGGCGGAGGAGCCGCAGCTGCTGCACGGTGCGGGCATCTGTAAG
                      *************** ********* ************ ** ************

                        16  17         18                19  20 21 n     22 23
LIN28P-Ch26     TGGTTCAACGTGCCCATGGGGTTCGGCTTCCTGTCCATGACCGCCTGCGCGGGGGTCGCC
LIN28           TGGTTCAACGTGCGCATGGGGTTCGGCTTCCTGTCCATGACCGCCCGCGCAGGGGTCGCG
                ************* ******************************* **** ********

                  24       25                                              26
LIN28P-Ch26     CTCGACCCCCCCGGTGGATGTCTTTGTGCACCAGAGTAAGCTGCACATGGAGGGCTTCCG
LIN28           CTCGACCCCCC-GGTGGATGTCTTTGTGCACCAGAGTAAGCTGCACATGGAGGGCTTCCG
                *********** ************************************************

                                    27                       28
LIN28P-Ch26     GAGCCTGAAGGAGGGGGAGGCCGTGGAGTTCACCTTTAAGAAGTCAGCCAAGGGCCTGGA
LIN28           GAGCCTGAAGGAGGGGGAGGCCGTGGAGTTCACCTTTAAGAAGTCCGCCAAAGGCCTGGA
                ********************************************* ***** ********

                       29      30                                   31
LIN28P-Ch26     ATCTCTCAGAGTCACTGGCCCTTGGG----------------------------------
LIN28           ATCTATCCGAGTCACCGGCCCTGGGGGGGTGTTCTGTATTGGGAGTGAAAGGCGGCCCAA
                **** ** ******* ****** ***

                                   32
LIN28P-Ch26     ------------------------------------------------------------
LIN28           AGGGAAGAATATGCAGAAACGCAGATCAAAGGGAGACAGGTGCTACAACTGTGGAGGTCT
```

Figure S3. Sequence alignment of *LIN28P-Ch:26* to the *LIN28* transcript. *LIN28P-Ch:26* sequenced region (top) is shown aligned to the matching region of the *LIN28* mRNA transcript (bottom). Matching nucleotides are indicated by a star below both nucleotides. CpG dinucleotides are highlighted in yellow on both sequences. Numbers above the pseudogene sequence indicates the placement of each CpG site in relation to the protein-coding region of the *LIN28* gene, with red numbers indicating the CpG sites analyzed in the pseudogene. CpG sites of pseudogenes that do not correspond to a CpG location within the *LIN28* parental gene are represented by 'n'. CpG dinucleotides highlighted in blue correspond to the CpG sites analyzed in the *LIN28* gene. Site 9 was identified as a likely SNP (C>T conversion), and thus was excluded from further analysis.

```
                26                    27                      28
LIN28P-Ch28     -----------------------------------CTTTAAGAAGTCCACCAAGGGCCTG
LIN28           CGGAGCCTGAAGGAGGGGGAGGCCGTGGAGTTCACCTTTAAGAAGTCCGCCAAAGGCCTG
                                                   ************* **** ******

                         29      30                                    31
LIN28P-Ch28     GAATCTATCCGAGTCACCGGCCCTGGTGGGGTGTTCTGTATTGAGAGTGAAAGGCGGCCC
LIN28           GAATCTATCCGAGTCACCGGCCCTGGGGGGGTGTTCTGTATTGGGAGTGAAAGGCGGCCC
                ************************** **************** ****************

                                     32                   n
LIN28P-Ch28     AAAGGGAAGAATATGCAGAAACACACATCAA-GGGAGACAGGCGCTACAACTGTGCAGGT
LIN28           AAAGGGAAGAATATGCAGAAACGCAGATCAAAGGGAGACAGGTGCTACAACTGTGGAGGT
                ********************** ** ***** ********** ************ ****

                           n                      33
LIN28P-Ch28     CTAGACCATCACGCCAAGGAATGCAAACTGCCACCCCAGCCCAAGAAGTGCCACTTCTGC
LIN28           CTAGACCATCATGCCAAGGAGTGCAAACTGCCACCGCAGCCCAAGAAGTGCCATTTCTGC
                *********** ******** ************** ***************** ******

                              n          34
LIN28P-Ch28     CAGAGCATCAGCCACGTGGTAGCCTCGTGCCCACTGAAGGCCCAGCAAGCTCCCAGCTCC
LIN28           CAGAGCATCAACCATATGGTAGCTTCGTGCCCACTGAAGGCCCAGCAAGCTCCCAGCTCC
                ********** ***   ******* ***********************************

                                      35
LIN28P-Ch28     CAGGGAAAGCCAGCCTACTTTCCGGAGGAGGAAGAAGAGATCCATAGCTCTGTCATGCTC
LIN28           CAGGGAAAGCCAGCCTACTTTCGGGAGGAGGAAGAAGAGATCCATAGCTCTGCCATGCTC
                ********************** ***************************** *******
```

Figure S4. Sequence alignment of *LIN28P-Ch:28* to the *LIN28* transcript. *LIN28P-Ch:28* sequenced region (top) is shown aligned to the matching region of the *LIN28* mRNA transcript (bottom). Matching nucleotides are indicated by a star below both nucleotides. CpG dinucleotides are highlighted in yellow on both sequences. Numbers above the pseudogene sequence indicates the placement of each CpG site in relation to the protein-coding region of the *LIN28* gene, with red numbers indicating the CpG sites analyzed in the pseudogene. CpG sites of pseudogenes that do not correspond to a CpG location within the *LIN28* parental gene are represented by ‘n’. CpG dinucleotides highlighted in blue correspond to the CpG sites analyzed in the *LIN28* gene.

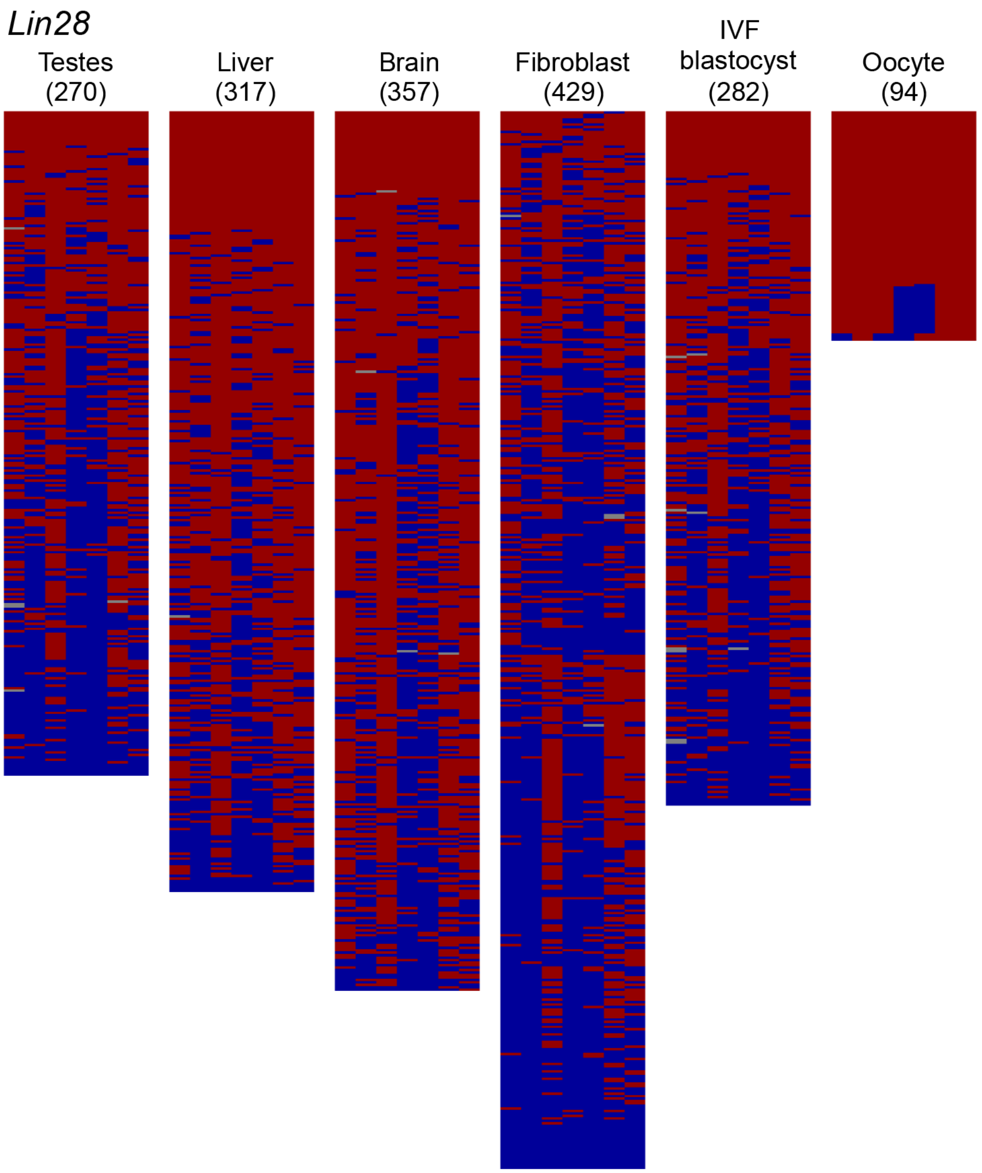

Figure S5. Heat map of *LIN28* individual reads from 454 sequencing. Each individual DNA molecule is represented for testes, liver, brain, fibroblast, IVF blastocyst, and oocyte. Red cells represent a methylated cytosine and blue cells represent an unmethylated cytosine. Gray cells represent a methylation read that was absent from sequencing. The total number of sequencing reads for a given tissue are indicated in parentheses below each tissue.

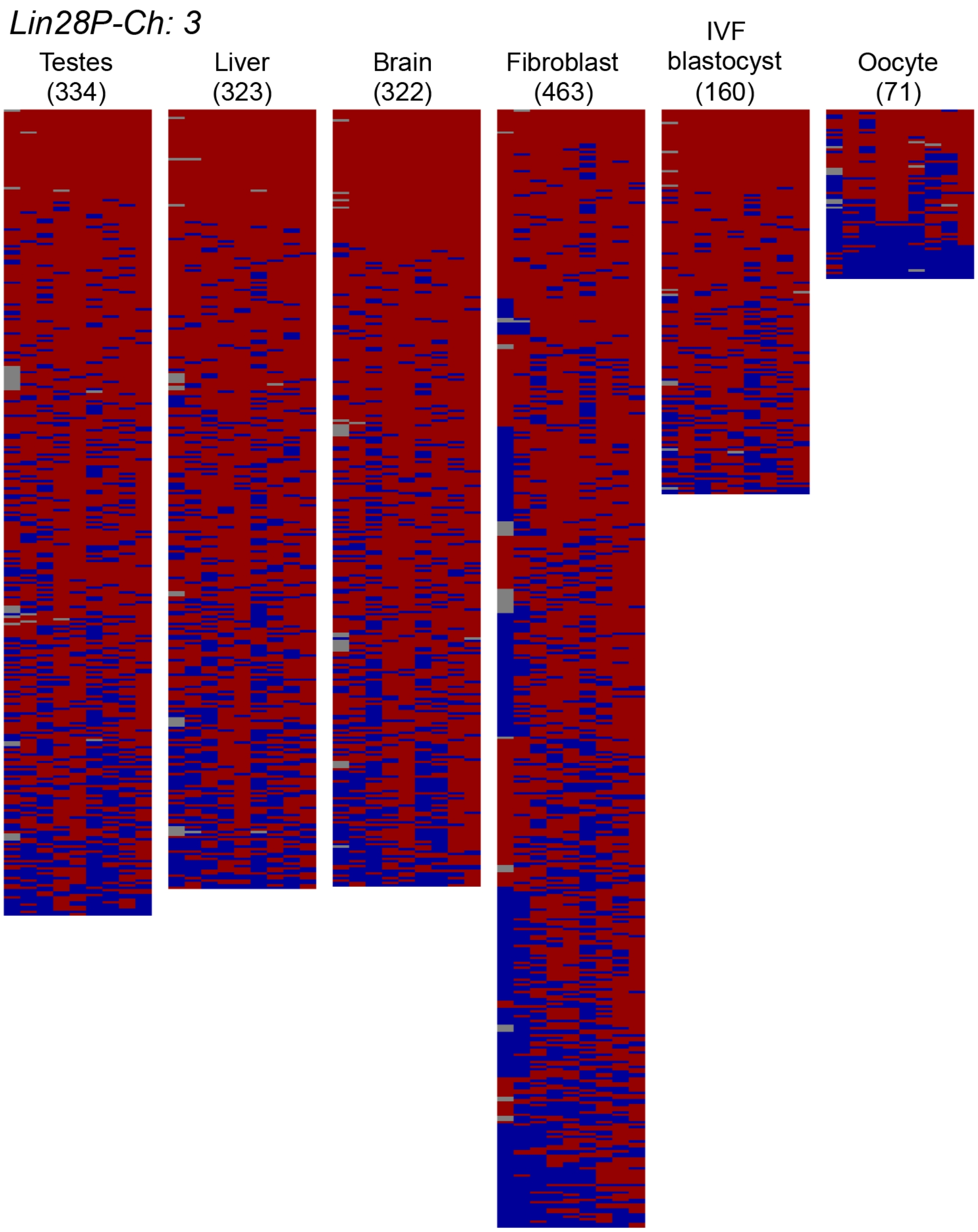

Figure S6. Heat map of *LIN28P-Ch:3* individual reads from 454 sequencing. Each individual DNA molecule is represented for testes, liver, brain, fibroblast, IVF blastocyst, and oocyte. Red cells represent a methylated cytosine and blue cells represent an unmethylated cytosine. Gray cells represent a methylation read that was absent from sequencing. The total number of sequencing reads for a given tissue are indicated in parentheses below each tissue.

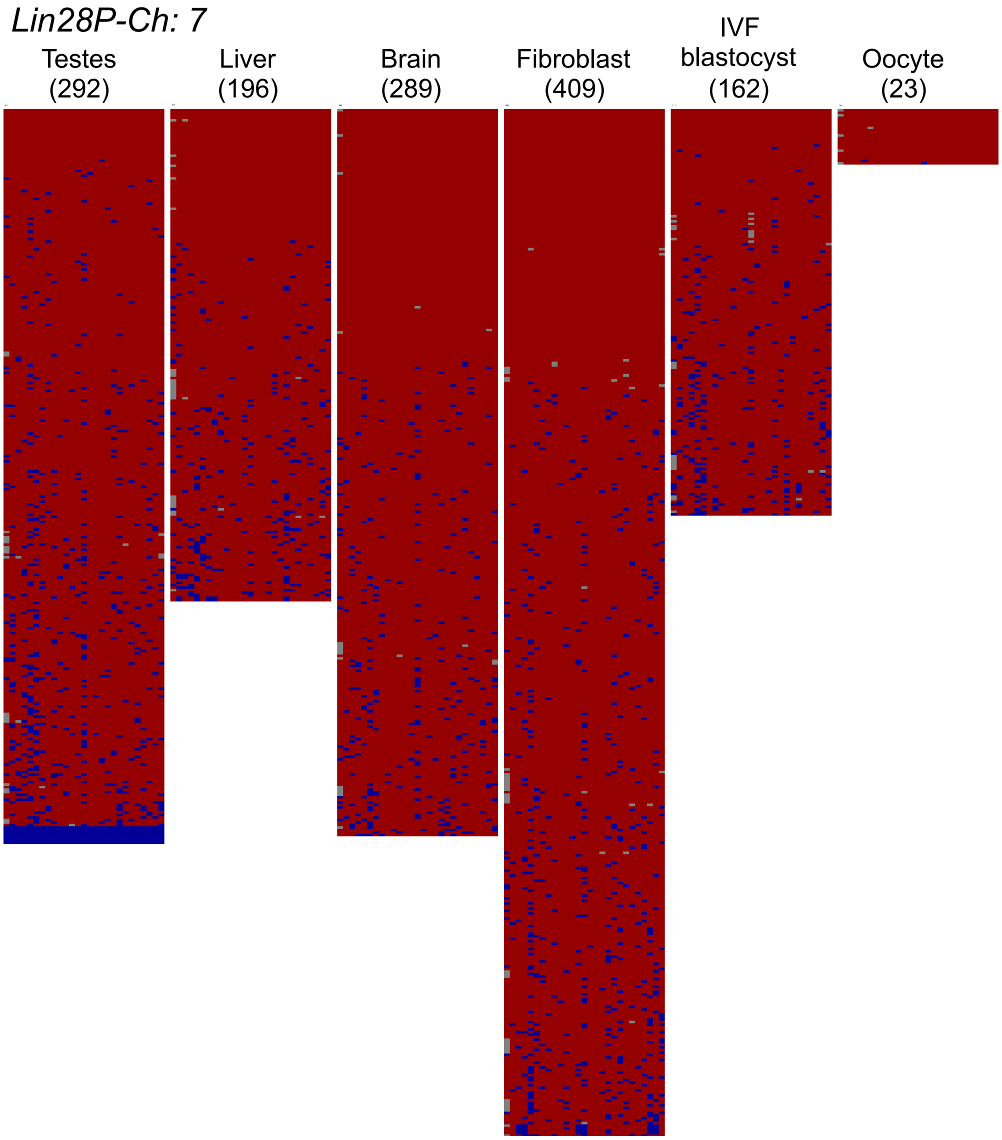

Figure S7. Heat map of *LIN28P-Ch:7* individual reads from 454 sequencing. Each individual DNA molecule is represented for testes, liver, brain, fibroblast, IVF blastocyst, and oocyte. Red cells represent a methylated cytosine and blue cells represent an unmethylated cytosine. Gray cells represent a methylation read that was absent from sequencing. The total number of sequencing reads for a given tissue are indicated in parentheses below each tissue.

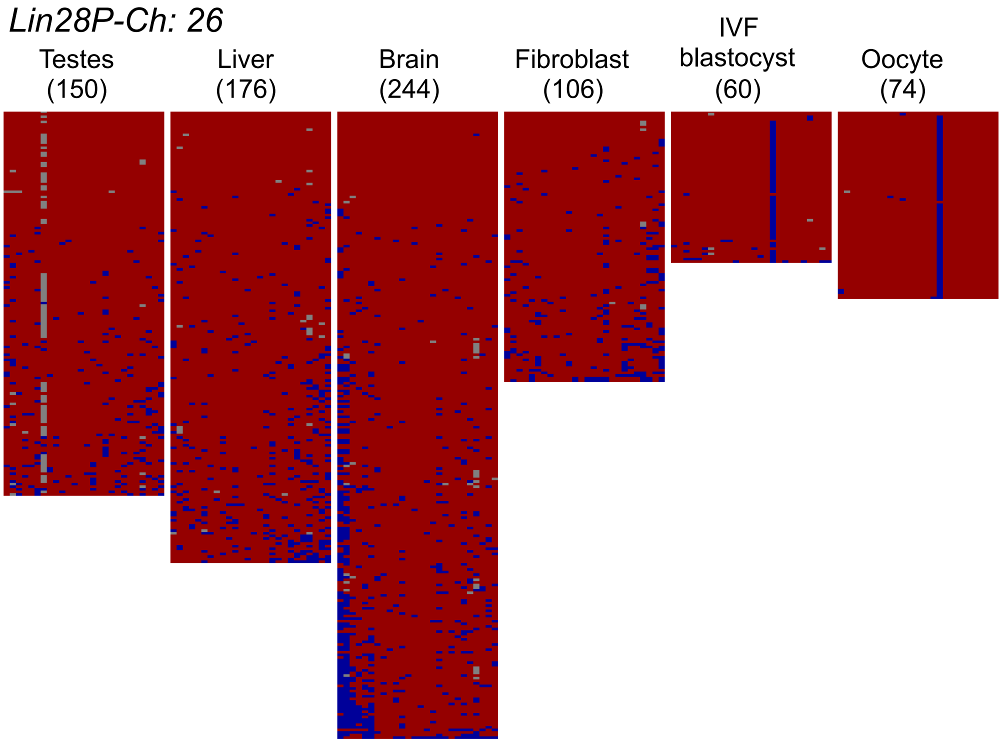

Figure S8. Heat map of *LIN28P-Ch:26* individual reads from 454 sequencing. Each individual DNA molecule is represented for testes, liver, brain, fibroblast, IVF blastocyst, and oocyte. Red cells represent a methylated cytosine and blue cells represent an unmethylated cytosine. Gray cells represent a methylation read that was absent from sequencing. The total number of sequencing reads for a given tissue are indicated in parentheses below each tissue.

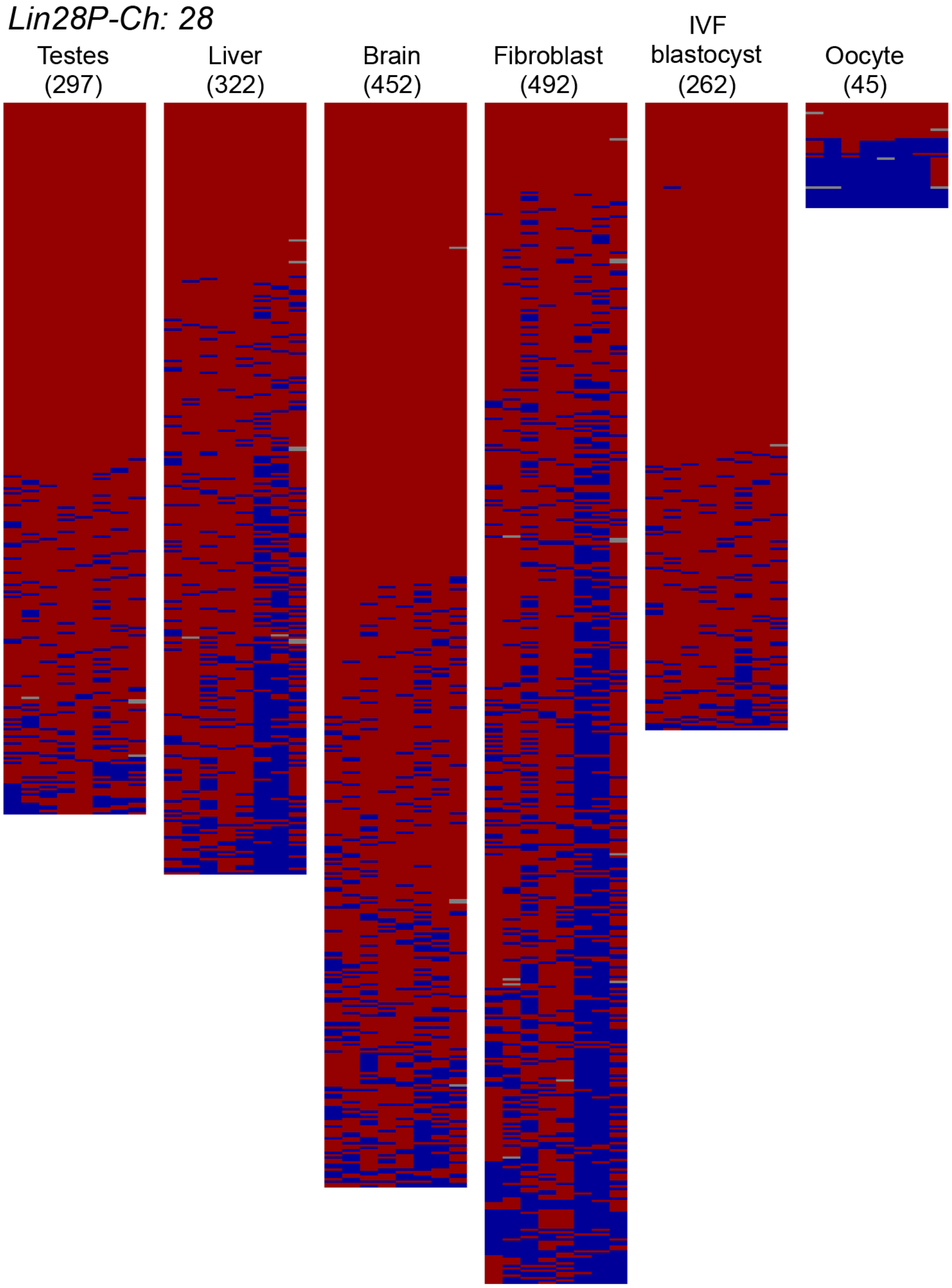

Figure S9. Heat map of *LIN28P-Ch:28* individual reads from 454 sequencing. Each individual DNA molecule is represented for testes, liver, brain, fibroblast, IVF blastocyst, and oocyte. Red cells represent a methylated cytosine and blue cells represent an unmethylated cytosine. Gray cells represent a methylation read that was absent from sequencing. The total number of sequencing reads for a given tissue are indicated in parentheses below each tissue.
